# Is it possible to automate the discovery of process maps for the time-driven activity-based costing method? A systematic review

**DOI:** 10.1186/s12913-023-10411-z

**Published:** 2023-12-13

**Authors:** Franciele Iachecen, Marcelo Rosano Dallagassa, Eduardo Alves Portela Santos, Deborah Ribeiro Carvalho, Sérgio Ossamu Ioshii

**Affiliations:** 1 Graduate Program in Health Technology, Pontifícia Universidade Católica do Paraná., 1155, Imaculada Conceição st., Curitiba, Paraná 80215-90 Brazil; 2 Department of Applied Social Sciences, Universidade Federal do Paraná, Curitiba, Paraná Brazil

**Keywords:** Time-driven activity-based costing, Process maps, Process mining, Health care management

## Abstract

**Objectives:**

The main objective of this manuscript was to identify the methods used to create process maps for care pathways that utilized the time-driven activity-based costing method.

**Methods:**

This is a systematic mapping review. Searches were performed in the Embase, PubMed, CINAHL, Scopus, and Web of Science electronic literature databases from 2004 to September 25, 2022. The included studies reported practical cases from healthcare institutions in all medical fields as long as the time-driven activity-based costing method was employed. We used the time-driven activity-based costing method and analyzed the created process maps and a qualitative approach to identify the main fields.

**Results:**

A total of 412 studies were retrieved, and 70 articles were included. Most of the articles are related to the fields of orthopedics and childbirth-related to hospital surgical procedures. We also identified various studies in the field of oncology and telemedicine services. The main methods for creating the process maps were direct observational practices, complemented by the involvement of multidisciplinary teams through surveys and interviews. Only 33% of the studies used hospital documents or healthcare data records to integrate with the process maps, and in 67% of the studies, the created maps were not validated by specialists.

**Conclusions:**

The application of process mining techniques effectively automates models generated through clinical pathways. They are applied to the time-driven activity-based costing method, making the process more agile and contributing to the visualization of high degrees of variations encountered in processes, thereby making it possible to enhance and achieve continual improvements in processes.

## Introduction

The time-driven activity-based costing (TDABC) method is a strategy for measuring costs to obtain the actual costs of a given process in healthcare [1, 2]. There are seven steps in the TDABC method [3]: (1) selecting the medical condition, (2) defining the clinical pathway, including the mapping of all key activities performed within the entire care cycle, (3) creating the clinical process maps included in each activity in the patient clinical pathway, incorporating all direct and indirect resources, (4) obtaining time estimates for each process for activities and resources used for each patient, (5) estimating the cost of using patient care resources, the cost of all direct and indirect resources involved in care delivery, (6) estimating the capacity of each resource and calculating the use cost rate and (7) calculating the total cost of patient care [3].

The TBADC method has recently expanded its use in healthcare in the past few years [2, 4, 5]. One of the main steps in applying the TDABC method consists of creating a process map describing all the activities in the analyzed clinical pathway and related resources the patient uses. Process maps assist in cost measurements by allocating resources in the analyzed clinical pathway and involving the necessary times for each process. In this context, the visualization of the involved costs becomes more tangible and can be calculated [6]. After creating the process maps, the costs of each activity are calculated to define the capacity unit value [5]. These activities can then be calculated jointly to measure the total cost of an entire service or care episode [7].

The TDABC method is considered the most precise method for measuring the costs of a specified clinical pathway due to its capability to measure the resources consumed by each patient based on two parameters: activity and time [8, 9]. It accurately provides expenditures based on process maps to navigate the steps a patient must complete throughout their care cycle [10]. In its traditional development method, the construction of clinical pathway process maps relies on conducting research and individual interviews with employees and experts in a particular field to estimate the percentage and time spent on an activity or by direct observation of the procedure [2].

The TDABC method has proven to be an effective tool for aiding administrators of healthcare initiatives based on value and performing micro-cost studies [2–4, 7]. The application of the method can also facilitate cost forecasting, supply information for improved management of resources and processes, and also as a basis for making future investment decisions and analyzing underused or unused capabilities in institutions [9]. A systematic review concluded that most studies on the TDABC method in healthcare institutions achieved results that contributed to cost savings. That same review also concluded that applying the TDABC method provided a truer perception to the participating members of the required resources and costs for performing each activity of the analyzed clinical pathways [11]. In addition, as TDABC relies on granular, procedure-level costs instead of broad categories, stakeholders are allowed to see what resources were used for what purposes [12].

There are even differences in the methodologies among the published studies due to the complexity of the healthcare field and the great diversity of costs and the activities present in healthcare services, which can impact cost forecasts [4]. The clinical and non-clinical teams supply information to enable the generation of process maps required for a service or procedure in the traditional TDABC method. Subsequently, the TDABC team is responsible for estimating the capacity of each resource and the necessary time based on surveys by institution professionals, who are specialists on the subject and directly observe the procedure [13]. A systematic review [3] showed the main collection methods for the TDABC method, which were derived from direct observation of procedures and interviews, meetings with the teams, or workshops. However, only one study mentioned the opportunity for creating automated process maps from the hospital data system [3]. The result from that review demonstrates the creation of process maps, even though they were prepared manually, which increases the required time for creating the process maps and the conclusion of the TDABC method.

Some systematic reviews were published on the subject [3, 11]; however, the publications still need to exploit which methods and tools were adopted to create the process maps essential for implementing the TDABC method. Therefore, systematic reviews addressing that topic are necessary for providing support to healthcare administrators regarding the possibility of automating the discovery of process maps to streamline part of the traditional process that can take weeks, according to some recent publications [14–19].

The main objective of this systematic review was to identify the methods used to create process maps for care pathways that utilized the time-driven activity-based costing method. That systematic review aimed to answer three questions: (1) What are the main healthcare fields applied to the TDABC method? (2) What methods were the most used for creating the process maps? (3) Have the created process maps been validated by specialists in the healthcare field?

## Methods

This systematic literature review was guided by the Preferred Reporting Items for Systematic Reviews and Meta-analyses (PRISMA) statement [20], following the recommendations from the Cochrane Handbook [21].

### Inclusion and exclusion criteria

The inclusion criteria were studies published in peer-reviewed journals related to practical cases at healthcare institutions independent from the medical field where the TDABC method was applied. Besides that, we selected studies that measured cost by the TDABC method. All study designs were considered. Literature reviews, abstracts, conference papers, letters to editors, editorials, and gray literature were excluded. We included studies that actively created process maps for applying the TDBAC method and studies that utilized existing process maps found in guidelines, clinical protocols, or established care pathways in the literature.

### Literature search strategy

Systematic searches were conducted on the following electronic databases: Embase, PubMed, CINAHL, Scopus, and Web of Science, including publications from 2004 to September 25, 2022. The search strategy was performed based on the following keywords: ((“time-driven activity based cost” OR “time-driven activity based costs” OR “time driven activity based cost” OR “time driven activity based costs” OR “timedriven activity based cost” OR “timedriven activity based costs” OR “time-driven cost” OR “time-driven costs” OR “time driven cost” OR “time driven costs” OR “timedriven cost” OR “timedriven costs” OR “TDABC”) AND (“Costs OR Cost Analysis”)). The searches were limited to full-text studies without language restrictions.

### The screening process of the studies

Two independent reviewers performed the study selection process (F.I. and M.R.D.). The selected studies were listed on two Microsoft Excel spreadsheets, abiding by the numerical and alphabetical order based on the first author and the publication year of the study. Each reviewer received the same spreadsheet, including the authors, publication year, title, complete abstract from the study, two columns for their decisions for registering the “yes or no” information, and a column for justification, where each reviewer filled in the reason for inclusion or exclusion of the study. In the first reviewing round, each reviewer analyzed the titles and abstracts, defining if the study would enter the step for reading the entire article. After concluding the independent review step, the selection results were compared. When any uncertainties on the aspects of content analysis occurred, the independent reviewers (F.I. and M.R.D.) discussed them until they agreed. In case of any disagreement, a third reviewer (D.R.C.) obtained consensus by discussing the studies or by arbitration.

### Data extraction

The independent authors (F.I. and M.R.D.) read the entire text from each study after the studies were selected. When they identified studies that were noncompliant with the eligibility criteria or were unrelated to practical cases for applying the TDABC method at healthcare institutions and did not measure costs by these methods, those studies were discarded. The eligible studies were listed on a Microsoft Excel spreadsheet after making the final selection, in numerical and alphabetical order, listed by the first author and the study publication year. The data collection was performed by grouping the studies according to their publication year, healthcare fields where the TDABC method was applied, and the construction method of the process maps for each study.

After selecting the studies included in this systematic review, the results were also independently generated by two authors (F.I. and M.R.D.). The results’ structure, organized into four spreadsheets, was devised, and filled out by each author. The results were compared upon completion to avoid errors in allocating final studies.

### The risk of bias in eligible studies

The risk of bias of eligible studies and the evaluation of their external validity was not performed, as there were no risk instruments for gauging specific risks of bias for the type of studies included in this review. However, a detailed evaluation of the studies enabled the identification of studies. In contrast, there was insufficient clarity in the methodological process to apply the TDABC method, thereby making it possible to use questionnaires and recommendations for future studies.

## Results

### Study selection and PRISMA flow diagram

**Fig. 1:**
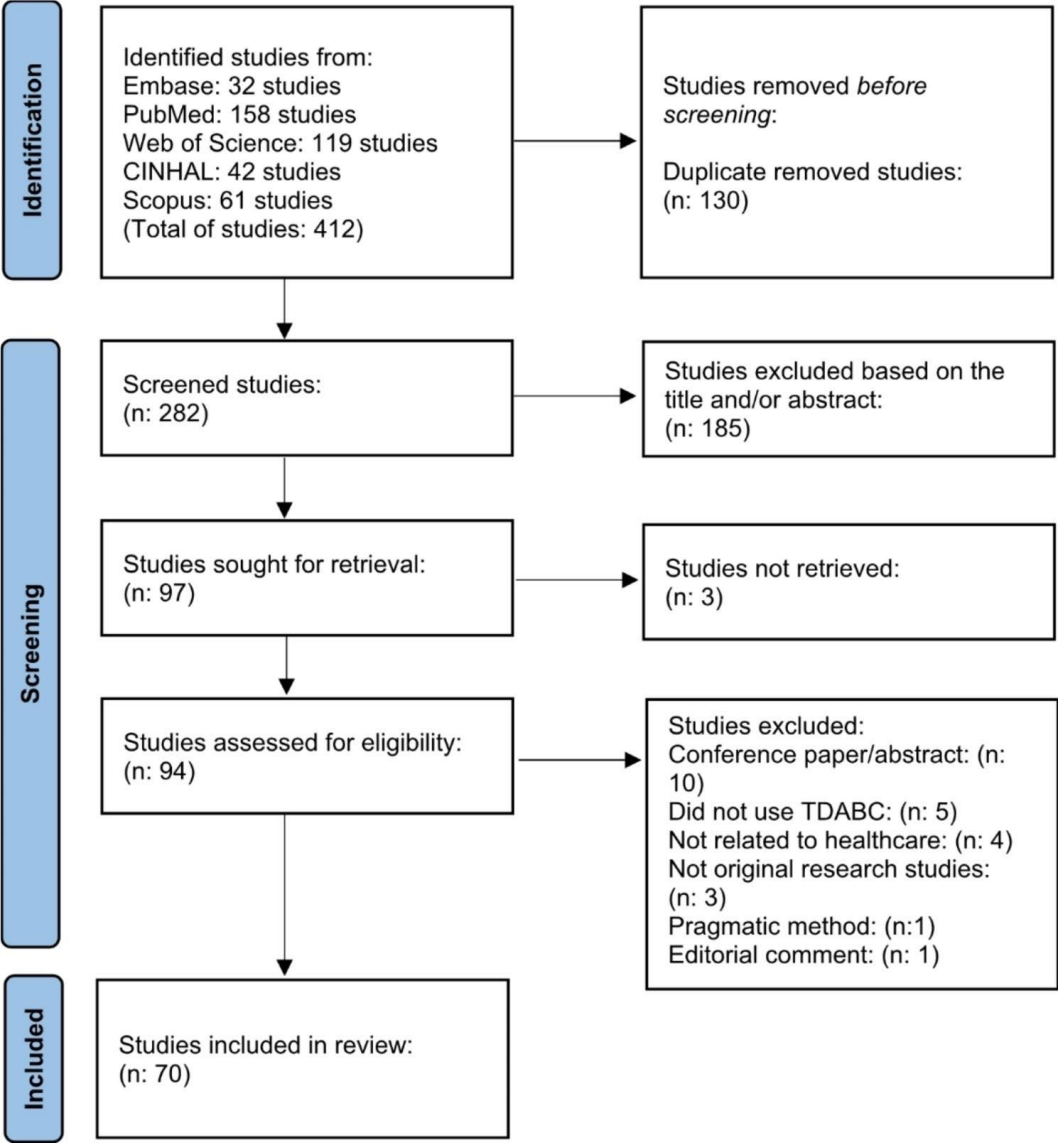
Flowchart of the selected studies according to the PRISMA statement

A total of 412 studies were found after searching the selected databases. We identified 130 duplicate studies; there were 282 remaining studies for analysis. Among those, 185 were excluded based on the title or abstract, as they did not mention the use of the TDABC method or were unrelated to the healthcare field. After reviewing the abstracts, 97 studies remained for analysis. Finally, a total of seventy articles were included in our study (Fig. 1).

### General study characteristics

The number of studies with publications related to the TDABC method in the healthcare field has increased in the recent past. Forty-three studies were identified from the 70 selected studies for this review from 2019 to 2022. The number represents 61.4% of the total studies included in the analysis, as shown in Table 1.

**Table 1:** The number of studies is based on their year of publication

Year of publication	Number of studies	(%)
2022	8	11.4
2021	15	21.4
2020	8	11.4
2019	12	17.1
2018	6	8.6
2017	5	7.1
2016	10	14.3
2015	4	5.7
2013	1	1.4
2011	1	1.4
Total	70	100.0

Table 2 shows the countries that have applied the TDABC method. Most of the studies are from the United States of America (62.8%), Brazil (7.1%), Australia (4.2%), the United Kingdom (4.2%), and Belgium (4.2%).

**Table 2:** The number of studies based on their country of application of the TDABC.

Country	Reference	Number of studies	(%)
United States of America	[2, 5, 14, 15, 18, 19, 22–59]	44	62.8
Brazil	[16, 60–63]	5	7.1
Australia	[6, 64, 65]	3	4.2
United Kingdom	[66–68]	3	4.2
Belgium	[69–71]	3	4.2
Canada	[72, 73]	2	2.8
Denmark	[74]	1	1.4
France	[75]	1	1.4
Zimbabwe	[76]	1	1.4
Ireland	[77]	1	1.4
Sweden	[78]	1	1.4
Finland	[79]	1	1.4
Pakistan	[80]	1	1.4
India	[81]	1	1.4
Germany	[82]	1	1.4
Rwanda	[83]	1	1.4

Subsequently, we analyzed the healthcare fields where the TDABC method was applied. There is a great variety of hospital surgical procedures, outpatient surgeries, miscellaneous types of hospital services, and sectors where the TDABC method was used for non-hospital services. The TDABC method was applied most commonly to knee arthroplasty (5 studies) in hospital surgical procedures [14, 50, 67, 74, 80], total hip arthroplasty (3 studies) [14, 50, 74], childbirth, and caesarean section (2 studies) [71, 83] and pediatric appendectomy/appendicitis (2 studies) [58, 59].

Diverse types of outpatient surgical procedures were performed. The procedures classified as a miscellaneous hospital or outpatient procedures were predominantly extracorporeal photopheresis procedures (2 studies) [62, 75] and red blood cell transfusion in patients with beta-thalassemia (2 studies) [64, 65]. The studies addressed diverse subjects on non-hospital services, predominantly on studies that proposed to evaluate telemedicine services (3 studies) [15, 51, 63].

We identified some studies in the field of oncology for different clinical pathways, such as breast cancer [31, 56], prostate cancer [30, 39, 42, 79], endometrial cancer [49], uterine cancer [55], and hepatocellular carcinoma [45]. Comparative studies involving new surgical methods through robotic intervention versus traditional methods were also uncovered in our search [44, 60]. All the identified fields are demonstrated in Table 3.

**Table 3:** Healthcare fields where the TDABC method has been applied

Health care fields	Articles
**Surgical conditions for hospital services**	
Knee arthroplasty	[14]
	[74]
	[67]
	[80]
	[50]
Total hip arthroplasty	[14]
	[74]
	[50]
Childbirth and cesarian	[71]
	[83]
Pediatric appendectomy and pediatric appendicitis	[58]
	[59]
Tibial plateau fractures	[22]
Robot-assisted laparoscopic pyeloplasty	[26]
Total shoulder arthroplasty	[28]
Interventional coronary procedures	[16]
Robotic‑assisted versus retropubic radical prostatectomy	[60]
Coronary artery bypass graft	[33]
Stroke treatment	[61]
Total ankle replacements	[19]
Cost comparison of in-suite versus portable tunneled femoral central line placements in children	[38]
Rotator cuff repair	[40]
Radical cystectomy	[41]
Cardiovascular disease patients in a super-specialty hospital	[81]
Treatments of small renal masses	[43]
Retroperitoneal versus transperitoneal robotic-assisted laparoscopic partial nephrectomy	[44]
Transarterial chemoembolization, transarterial radioembolization, and ablation for hepatocellular carcinoma	[45]
Interventional radiology for image-guided vascular malformation treatment	[82]
Acute urolithiasis cycle	[46]
Isolated ankle fractures	[47]
Urologic stone disease	[52]
Fractures of the proximal humerus	[68]
Intraoperative radiation therapy for early-stage breast cancer	[56]
**Surgical conditions for outpatient services**	
Endoscopic sinus surgery	[72]
Adenotonsillectomy	[23]
Vitrectomy	[24]
Anticoagulation therapy	[25]
Anesthesia care costs for outpatient oncology surgery	[34]
Endoscopic versus open carpal tunnel release	[18]
Cataract surgery	[73]
**Miscellaneous hospital or outpatient services**	
Extracorporeal photopheresis	[75]
	[62]
Red blood cell transfusion in patients with beta-thalassemia	[64]
	[65]
Abdomen and pelvis computed tomography	[5]
Operating room efficiency	[6]
Radiation treatment options for spinal metastases	[27]
Sentinel node biopsy in solid head and neck tumors	[69]
Health Economics in Radiation Oncology (ESTRO-HERO) project	[70]
Radiologic occult injury screening	[29]
Type 2 diabetes mellitus	[77]
Altered fractionation in early-stage breast cancer	[31]
Treating depression using internet-based cognitive-behavioral therapy	[32]
Managing recurrent urinary tract infections	[35]
Costs of ultrasound versus magnetic resonance imaging	[36]
Stereotactic body radiation therapy of primary pancreatic cancer	[37]
Cost of care in benign prostatic hyperplasia	[2]
Magnetic resonance imaging and computed tomography in prostate cancer	[79]
Short- and long-term costs of treating localized, low-risk prostate cancer	[42]
Suspected pediatric midgut volvulus using either fluoroscopic upper gastrointestinal examination or focused abdominal ultrasound	[48]
Adjuvant radiation therapy for high- to intermediate-risk endometrial cancer	[49]
Oral contrast administration in the emergency department	[53]
Adenotonsillectomy	[54]
Adjuvant vaginal cuff brachytherapy for uterine cancer in an integrated brachytherapy	[55]
Low-dose-rate and high-dose-rate brachytherapy for low-risk prostate cancer	[39]
Prostate brachytherapy	[84]
**Non-hospital services**	
Physical activity care pathway program	[66]
A national tuberculosis control program	[76]
Telehealth ostomy self-management training	[15]
Reimbursement of prostate cancer radiation therapy	[30]
Patients with multiple chronic conditions	[78]
Telemedicine services in radiation oncology	[51]
Telemedicine service in ophthalmology	[63]

### Process map construction

We analyzed how the process maps were created in the selected studies, as the process map creation is an important step for the preparation and application of the TDABC method based on the following five perspectives: (1) direct observation of the procedure; (2) was any hospital documentation and/or medical records used?; (3) did any research, involvement, interview, or meetings with clinical staff occur?; (4) were data from electronic health records used?; (5) were the process maps validated? were other types involved in constructing the process maps?

Most of the included studies utilized direct observations of the procedures [74] and involved the clinical staff, specialists, and multidisciplinary team to create the process maps [73]. 33 (47.1%) of the studies reported using data from electronic healthcare records or hospital documentation to complement the construction of the maps. On the other hand, the majority [67] of the analyzed studies did not validate the process maps after being concluded. The method utilized for creating the process maps in three studies was classified as unclear in their publications; thus, they were classified that way [19, 30, 70].

Regarding the other types of construction, we identified the process maps of a study performed in Canada that created a model for a clinical pathway of a patient submitted to a routine outpatient endoscopic sinus surgery for estimating the cost of this procedure on the patient [72]. In a study performed in Brazil, five main perspectives were analyzed, and the process maps were complemented by the clinical analysis of the protocols in the ischemic stroke condition [61]. The details of how the process maps were constructed in each study are shown in Table 4.

**Table 4:** The methods used for creating the process maps for implementing the TDABC.

Study	Direct observation of the procedure	Were any hospital documentation and/or medical records used?	Did research, involvement, interviews, or meetings with clinical staff occur?	Were data from electronic health records used?	Were the process maps validated?	Other types were used for constructing the process maps
Akhavan, Ward et al. 2016 [14]	Yes	Yes	-	-	Yes	-
Albright, Only et al. 2022 [22]	-	Yes	-	Yes	Yes	-
Andreasen, Holm et al. 2017 [74]	Yes	Yes	Yes	Yes	-	-
Anzai, Heilbrun et al. 2017 [5]	Yes	-	Yes	Yes	-	-
Au and Rudmik 2013 [72]	-	-	-	-	-	A clinical pathway model was created for patients undergoing routine outpatient endoscopic sinus surgery.
Azar, Leblond et al. 2017 [75]	Yes	Yes	-	Yes	Yes	-
Balakrishnan, Goico et al. 2015 [23]	Yes	-	Yes	-	Yes	-
Basto, Chahal et al. 2019 [6]	Yes	-	-	-	-	-
Berkowitz, Sternberg et al. 2021 [24]	Yes	-	-	Yes	-	-
Bobade, Helmers et al. 2019 [25]	Yes	Yes	-	Yes	-	-
Bodar, Srinivasan et al. 2020 [26]	Yes	-	Yes	Yes	Yes	-
Boehler, Milton et al. 2011 [66]	-	-	Yes	Yes	Yes	-
Boyce-Fappiano, Ning et al. 2020 [27]	Yes	-	Yes	-	-	-
Burns, Haysom et al. 2019 [64]	Yes	Yes	Yes	-	Yes	-
Carducci, Mahendraraj et al. 2021 [28]	Yes	Yes	Yes	-	-	-
Chen, Sabharwal et al. 2015 [67]	Yes	-	-	-	-	-
Chirenda, Nhlema Simwaka et al. 2021 [76]	Yes	-	Yes	-	-	-
Cidav, Marcus et al. 2021 [15]	Yes	-	Yes	-	Yes	-
Crott, Lawson et al. 2016 [69]	-	-	Yes	-	-	-
da Silva Etges, Cruz et al. 2022 [16]	Yes	Yes	Yes	Yes	Yes	-
de Oliveira, Guimaraes et al. 2021 [60]	Yes	Yes	-	-	-	-
Defourny, Perrier et al. 2019 [70]	-	-	-	-	-	Unclear
Deutsch, Zomorrodi et al. 2022 [29]	-	Yes	Yes	Yes	-	-
Doyle, O’Donnell et al. 2022 [77]	-	Yes	Yes	Yes	Yes	-
Dubron, Verschaeve et al. 2021 [71]	Yes	-	Yes	-	-	-
Dutta, Bauer-Nilsen et al. 2018 [30]	-	-	-	-	-	Unclear
Dziemianowicz, Burmeister et al. 2021 [31]	Yes	-	Yes	-	-	-
El Alaoui and Lindefors 2016 [32]	Yes	-	Yes	Yes	-	-
Erhun, Mistry et al. 2015 [33]	Yes	-	Yes	-	Yes	-
Etges, Marcolino et al. 2022 [61]	Yes	Yes	Yes	Yes	Yes	Clinical protocols were used for the construction of the process models
French, Guzman et al. 2016 [34]	Yes	-	Yes	Yes	-	-
Gaitonde, Malik et al. 2018 [35]	Yes	-	Yes	-	-	-
Hagedorn, Hayatghaibi et al. 2019 [36]	Yes	-	Yes	Yes	-	-
Hamid, Matson et al. 2017 [19]	-	-	-	-	-	Unclear
Hawranko, Sohn et al. 2022 [37]	-	Yes	-	-	-	-
Hayatghaibi, Chau et al. 2020 [38]	Yes	-	Yes	-	Yes	-
Ilg, Laviana et al. 2016 [39]	Yes	-	Yes	-	Yes	-
Kaplan, Agarwal et al. 2015 [2]	-	-	Yes	-	-	-
Keel, Muhammad et al. 2020 [78]	Yes	Yes	Yes	Yes	Yes	-
Keyrilainen, Sjoblom et al. 2021 [79]	Yes	-	-	-	-	-
Khan, Albutt et al. 2019 [80]	Yes	-	Yes	-	Yes	-
Koehler, Balakrishnan et al. 2019 [18]	Yes	-	Yes	-	Yes	-
Koolmees, Ramkumar et al. 2022 [40]	-	Yes	-	-	-	-
Kukreja, Seif et al. 2021 [41]	Yes	-	-	-	-	-
Kumar, Siddharth et al. 2022 [81]	Yes	-	Yes	-	-	-
Laviana, Ilg et al. 2016 [42]	-	Yes	Yes	-	-	-
Laviana, Kundavaram et al. 2016 [43]	-	-	Yes	Yes	-	-
Laviana, Tan et al. 2018 [44]	-	-	Yes	-	-	-
Ljuboja, Ahmed et al. 2021 [45]	Yes	-	Yes	-	-	-
Masthoff, Schneider et al. 2021 [82]	Yes	-	Yes	Yes	-	-
McClintock, Friedlander et al. 2021 [46]	Yes	-	Yes	-	Yes	-
McCreary, White et al. 2018 [47]	Yes	Yes	Yes	Yes	-	-
McQuilten, Higgins et al. 2019 [65]	Yes	-	Yes	-	Yes	-
Nguyen, Sammer et al. 2020 [48]	Yes	Yes	Yes	Yes	Yes	-
Ning, Klopp et al. 2019 [49]	-	-	Yes	-	-	-
Odhiambo, Ruhumuriza et al. 2019 [83]	Yes	Yes	Yes	-	-	-
Palsis, Brehmer et al. 2018 [50]	Yes	-	Yes	-	-	-
Parikh, Chang et al. 2021 [51]	-	-	Yes	-	-	-
Pollard, Laviana et al. 2018 [52]	Yes	Yes	-	-	-	-
Sabharwal, Carter et al. 2016 [68]	-	-	Yes	-	Yes	-
Sadri, Sinigallia et al. 2021 [73]	Yes	-	Yes	-	-	-
Shankar, Parikh et al. 2019 [53]	Yes	Yes	Yes	-	-	-
Simmonds, Hollis et al. 2019 [54]	Yes	-	-	-	-	-
Su, Dutta et al. 2020 [55]	Yes	-	Yes	-	-	-
Suralik, Rudra et al. 2020 [56]	Yes	-	Yes	-	-	-
Thaker, Pugh et al. 2016 [84]	Yes	-	Yes	Yes	-	-
Vargas, Pereira et al. 2021 [62]	Yes	-	Yes	-	-	-
Yu, Abbas et al. 2016 [58]	Yes	Yes	Yes	Yes	Yes	-
Yu, Abbas et al. 2017 [59]	Yes	Yes	-	Yes	-	-
Zanotto, Etges et al. 2020 [63]	Yes	-	Yes	-	Yes	-

## Discussion

The objective of this review has been to analyze the main methods employed in creating process maps for applying the TDABC method, searching for answers to three key questions: (1) What are the main healthcare fields where the TDABC method is applied? (2) What methods were mostly used for creating the process maps? (3) Are the created process maps validated by specialists in those fields? Seventy studies were included. The most identified hospitalized surgical procedures were in the orthopedic field, and studies performed in oncology or procedures related to that field. Studies were comparing new technologies to traditional methods found in this review. We found three studies that addressed telemedicine services in non-hospital services.

The most studies are from the United States of America, however, other countries, such as Brazil, have also begun to adopt the method for cost mapping [16, 60–63]. Despite the increasing use of TDABC in health-related studies, methodological heterogeneity still exists in how researchers map their clinical processes, present their methods, and report their results [85]. We observed a wide variety of clinical pathways and other studied procedures. We identified a total of 63 different applications that utilized the TDABC method out of the total 70 included studies. This illustrates the significant heterogeneity in themes and applications in using TDABC. Despite having a high degree of heterogeneity (99.1%), this review demonstrated that the TDABC method can be applied to various conditions, allowing cost measurement through the best and most efficient method for cost measurement in healthcare [8, 9].

The main methods for creating process maps are performed by direct observation of the procedure with the involvement of specialists in those fields, and the participation of a multidisciplinary team. We found validation reports only in 33% of the studies among the created process maps. Regarding the tools used to build process maps, we identified studies using QlikView and R software [78], Microsoft Excel and Adobe Illustrator [82] and using digital platforms such as Bizagi or Microsoft Visio [86]. Regarding the use of electronic data in healthcare, one systematic review in 2017 identified a study where the process maps were created based on electronic medical records involving surgeries and medical care procedures [3]. In our review, the electronic medical records were used to construct the process maps identified in 23 studies [5, 16, 22, 24–26, 29, 32, 34, 36, 43, 47, 48, 58, 59, 61, 66, 74, 75, 77, 78, 82, 84], proving that using medical data from electronic medical records has enhanced and provided support to the process.

Although the use of electronic data has increased in the past few years, the step of creating process maps for the TDABC method still demands time and the availability of the multidisciplinary team when. We found studies that reported that the total period for constructing process maps ranged from one to 12 weeks [14, 18, 59, 66]. Since there is a growing number of studies utilizing electronic medical data and the construction of the process maps demands longer periods until they are concluded, applying process mining methods can supply new and efficient ways for mapping healthcare processes [3, 87]. Process mining aims to extract knowledge from data in electronic records [88, 89]. Process mining can generate healthcare flowcharts based on administrative or clinical data collected routinely, thereby enabling the automated identification of process maps by identifying events that generally occur in a time sequence [90]. A systematic review identified that healthcare is currently the main field of application for process mining, demonstrating its total applicability to this segment [91]. Another recent publication demonstrated that process mining provides the greatest applicability in the discovery for analyzing process models for evaluating patient healthcare, compliance process evaluation to compare medical protocols and clinical guidelines and performance, bottlenecks, and time management evaluations [92].

The main product process mining is capable of supplying, and it contributes to streamlining the construction of process maps for the TDABC method, which are care flow maps and workflow networks that describe the detailed flow of patients [93]. In care flow maps, the main activities are viewed based on their frequency and established relations between activities and their reoccurrences. The application of process mining also enables identifying the duration between activities [94]. In the case of the TDABC method, the direct timed observations can be automated by applying this technique, as the process maps can be generated in an automated manner through electronic data contained in the hospital data systems [95].

Healthcare processes are known to be highly dynamic, complex, and increasingly multidisciplinary [89]. Therefore, it is important to define the analysis goal to overcome this challenge, starting from the first step of TDABC by selecting the medical condition. After the selection, data processing can be carried out to reduce the variability of data in a patient’s event trajectory, discarding irrelevant information that may be present in the electronic health record. Following data processing, process mining techniques can be applied to generate a process flow, which can then be validated by experts in the field [92].

Process mining has a high potential to substantially contribute to creating process maps for preparing the TDABC method, streamlining the direct observations of procedures for measuring periods, which are currently performed manually. It is worth mentioning that applying process mining and discovering process models do not rule out the validity of specialized professionals in this field, nor the involvement of a multidisciplinary team at institutions. There were 23 studies in our review that validated process maps [14–16, 18, 22, 23, 26, 33, 38, 39, 46, 48, 58, 61, 63–66, 68, 75, 77, 78, 80].

Process mining could add different sources of information and merge them with the conventional model to minimize the biases from registering and validating the discovered maps. That would make it feasible to identify more precisely the fields with greater variability in processes [3] and aid healthcare administrators in identifying important markers of the process map, enabling improved and more agile process management. In future studies, we intend to develop the TDABC method in different oncology fields, utilizing process mining to prepare the process maps to calculate the cost of clinical pathways most relevant to the public healthcare field in Brazil.

## Conclusions

We identified that the TDABC method has been more commonly implemented in the past few years and applied to diverse healthcare fields, predominantly in hospital surgical services in orthopedics, oncology, and outpatient telemedicine services. We consider creating process maps, a fundamental step for preparing the TDABC method, which is still performed mostly conservatively. We believe there is great potential in applying process mining to contribute to that process due to its high degree of applicability in the healthcare field, as well as its capability to identify through the discovery of models the main activities, jointly with their established relations, as well as demonstrate the necessary time between one activity and another.

A high degree of variability is encountered in the healthcare field, and there is an increasing amount of data in hospital systems. It can be challenging to create manual process maps. Process mining is an effective technique that has displayed great potential for discovering healthcare clinical pathways, even when facing the large volume of information and the large variation of activities found in clinical pathways. The TDABC method would make this process more agile, minimize the need for analyzing the period of procedures, meetings, and workshops with key stakeholders in institutions, and aid healthcare administrators in improving their process analyses.

## Data Availability

All data generated or analyzed during this study are included in this published article.

## List of abbreviations

TDABC: time-driven activity-based costing
